# Assessment of Completeness of Hospital Readmission Rates Reported in Medicare Advantage Contracts’ Healthcare Effectiveness Data and Information Set

**DOI:** 10.1001/jamanetworkopen.2020.3555

**Published:** 2020-04-28

**Authors:** Daeho Kim, Rajesh Makineni, Orestis A. Panagiotou, Amal N. Trivedi

**Affiliations:** 1 Department of Health Services, Policy, and Practice, Brown University, Providence, Rhode Island; 2 Providence VA Medical Center, Providence, Rhode Island

## Abstract

This cross-sectional study evaluates the agreement between readmission rates reported by Medicare Advantage contracts and readmission rates calculated from their encounter data in the Healthcare Effectiveness Data and Information Set (HEDIS).

## Introduction

The Centers for Medicare & Medicaid Services (CMS) pays $6 billion in annual bonus payments to Medicare Advantage (MA) contracts that achieve 4 or more stars on a 5-star quality rating system.^1^ The CMS derives star ratings from 46 measures, including a 30-day hospital readmission measure reported by MA contracts to the Healthcare Effectiveness Data and Information Set (HEDIS). Limited analyses exist regarding the accuracy of reporting of MA contracts’ HEDIS quality data.^2,3^ Using CMS’s first-ever released MA encounter data, we assessed the agreement between the readmission rates reported by MA contracts and readmission rates calculated from their encounter data.

## Methods

This cross-sectional study was approved by Brown University’s institutional review board with a waiver of informed consent because of the infeasibility of acquiring consent for deidentified data. This study follows the Strengthening the Reporting of Observational Studies in Epidemiology (STROBE) reporting guideline.

We applied the HEDIS and CMS’ hospitalwide readmission specifications^4^ to MA encounter data and identified eligible index hospital admissions between January 1, 2015, and December 1, 2015. Index hospitalizations were defined as those for any condition accompanied by a discharge to home or a nonacute setting. Consistent with CMS’ specifications, we excluded admissions for medical treatment for cancer and discharges against medical advice. The study population included Medicare beneficiaries aged 65 years and older who had been continuously enrolled in an MA contract for 12 months before and 30 days after the index admission. We linked index admissions to patient-level HEDIS data reported by MA contracts. We assessed the completeness of the HEDIS-reported admissions and compared 30-day readmission rates calculated from encounter data against the contract-reported HEDIS readmission rates for the same patients. We calculated the star ratings of each MA contract on the basis of the 2017 cut points, which correspond to the 2015 data, of the readmission measure (5 stars, ≤8%; 4 stars, >8% to 10%; 3 stars, >10% to 12%; 2 stars, >12% to 15%; 1 star, >15%) using both the HEDIS-reported and encounter-based readmission rates. We then examined how each contract’s completeness of HEDIS-reported admissions was associated with its star ratings.

All statistical tests were conducted using 2-sided *t* tests, with significance set at *P* < .05. Data analyses were conducted using Stata statistical software version 15 (StataCorp). Data were analyzed from May 2019 to February 2020.

## Results

Among 1 175 341 index admissions (mean [SD] age, 77.6 [7.8] years; 512 377 [43.6%] male) from 441 MA contracts, 164 871 (14%) did not appear in HEDIS (Table). Among index admissions present in HEDIS, the readmission rate calculated from the encounter data was similar to the HEDIS-reported readmission rate (11.5% [95% CI, 11.3%-11.7%] vs 11.7% [95% CI, 11.5%-11.9%]; *P* = .31). Among index admissions that were not present in HEDIS, the readmission rate was higher than the HEDIS-reported readmission rate by 11.9 percentage points (23.6% [95% CI, 22.4%-24.8%] vs 11.7% [95% CI, 11.5%-11.9%]; *P* < .001). Overall, the readmission rates calculated from encounter data were higher than the HEDIS-reported rates (13.2% [95% CI, 12.8%-13.6%] vs 11.7% [95% CI, 11.5%-11.9%]; *P* = .004).

**Table.** Thirty-Day Readmission Rates for Medicare Advantage Enrollees^a^

Variable	Index admissions reported in HEDIS		
	No	Yes	Total
Index admissions present in encounter data, No.	164 871	1 010 470	1 175 341
Readmission rate, % (95% CI)			
In encounter data	23.6 (22.4-24.8)	11.5 (11.3-11.7)	13.2 (12.8-13.6)
In HEDIS	NA	11.7 (11.5-11.9)	NA

Abbreviations: HEDIS, Healthcare Effectiveness Data and Information Set; NA, not applicable.

^a^ Table shows 30-day hospital readmission rates in 2015 for Medicare Advantage enrollees aged 65 years or older after their previous hospitalization (ie, index admissions) reported from 441 Medicare Advantage contracts. The inclusion criteria for hospitalwide admission measure developed by the Centers for Medicare & Medicaid Services for the Hospital Readmissions Reduction Program were used.

Among the 441 MA contracts, the median proportion of index admissions that were missing in HEDIS was 13.1% (range, 0% to 74.8%; interquartile range, 7.9% to 20.0%). The difference between the HEDIS-reported readmission rates and the readmission rates calculated from encounter data ranged from −33.8% to 33.3% (interquartile range, −1.9% to 0.6%). The proportion of missing index admissions in HEDIS and the difference in readmission rates observed in HEDIS and encounter data were correlated (Pearson correlation coefficient, −0.40; 95% CI, −0.47 to −0.32) (Figure). Medicare Advantage contracts with less-complete reporting of admissions in HEDIS had a greater gain in their star ratings from readmission rates when using HEDIS data rather than encounter data (Figure). Among the 109 MA contracts with more than 20% of missing admissions (the 75th percentile of proportion of missing admissions) in HEDIS, 58 contracts gained 1 or more star ratings (1-star gain for 32 contracts; ≥2-star gain for 26 contracts) using the HEDIS-reported readmission rate compared with the rate observed in the contract’s encounter data.

**Figure.**
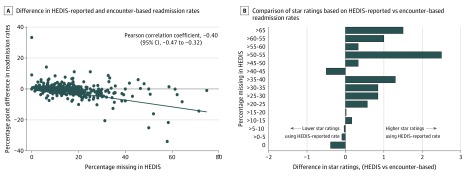
Medicare Advantage Contracts’ Healthcare Effectiveness Data and Information Set (HEDIS)–Reported and Encounter-Based Readmission Rates by Percentage of Missing Admissions in HEDIS A, Difference between HEDIS-reported and encounter-based readmission rates. B, Comparison of star ratings based on HEDIS-reported vs encounter-based readmission rate.

## Discussion

To our knowledge, this is the first validation study of the HEDIS quality readmission measure using MA encounter data. We found that readmission rates were statistically significantly higher in encounter data than in HEDIS data. This difference was associated with the underreporting of index admissions in HEDIS. Notably, the readmission rates of underreported admissions were approximately 2 times greater than the readmission rates of those included in HEDIS data. The proportion of underreported index admissions in HEDIS varied widely across MA contracts. Medicare Advantage contracts with the most complete reporting were penalized in rankings compared with contracts with less-complete HEDIS data. Limitations of this study include the assessment of a single quality measure and the use of 1 year of encounter data. Because HEDIS performance affects star ratings, bonus payments, and patients’ plan choices, CMS should consider rigorous audits of HEDIS data, particularly their denominators.
